# Application of a Successful Germ Cell Tumor Paradigm to the Challenges of Common Adult Solid Cancers

**Published:** 2021

**Authors:** Shi-Ming Tu, Matthew Campbell, Amishi Shah, Christopher J. Logothetis

**Affiliations:** Department of Genitourinary, Medical Oncology University of Texas MD Anderson Cancer Center, 1155 Pressler Street Houston, TX 77030-372, United States

**Keywords:** Cancer stem cell, Drug development, Personalized care, Precision medicine, Targeted therapy, Testicular Cancer

## Abstract

When we aspire to cure cancer, we may need to search no further than a curable cancer, such as Germ Cell Tumor of the Testis (TGCT). After all, a germ cell is a primordial stem cell. Importantly, TGCT provides a classic stem cell model of cancer that teaches us some invaluable lessons about curing other intractable solid tumors.

The intrinsic intratumoral heterogeneity of TGCT alludes to its stem-ness origin and nature. Which implicates the existence of putative lethal TGCT subtypes-the identification and detection of which may further enhance the cure rate and improve the therapeutic ratio of TGCT.

In this Mini review, we discuss about the role of biologic insights, clinical lessons, and therapeutic strategies in drug and therapy development. We illustrate some clinical pearls and perils when it concerns drug versus therapy development in the cure and care of patients with TGCT.

In many respects, we have cured more TGCT patients when we apply multimodal therapy rather than targeted therapy and integrated medicine rather than precision medicine. In principle and in practice, this is the implication of therapy versus drug development in improving the overall outcome and cure rate of patients with cancer.

## INTRODUCTION

Prior to the development of effective therapies, a diagnosis of metastatic germ cell tumor of the testis (TGCT) was invariably fatal [1]. The remarkable success that leads to a high cure rate for patients with TGCT irrespective of tumor volume is a singular milestone in the development of therapy for adult solid tumors [2-4]. Since then, many have strived to understand and replicate the TGCT experience in other cancers. By looking back at this sustained international effort, we may be able to apply its lessons to other solid tumors.

Unlike chronic myelogenous leukemia, TGCT is a heterogeneous cancer whose therapeutic success cannot be attributed to the remedy of a single molecular aberration in a malignant tumor [1,5,6]. Therefore, unlike chronic myelogenous leukemia, TGCT should be the norm, rather than an exception, that establishes a broadly applicable therapeutic paradigm for the vast majority of heterogeneous cancers when it concerns utility of targeted therapy versus multimodal therapy and feasibility of precision medicine versus integrated medicine in the personalized care and potential cure of common adult solid cancers.

In this article, we describe our shared view on how to apply the necessary but individually insufficient components as a rational therapeutic strategy modeled on the approach that led to the remarkable clinical achievement in the cure and care of patients with TGCT. These components include: 1) informative clinicopathologies, 2) effective interventions (surgery, radiation, chemotherapy), and 3) predictive biomarkers that allow therapy development to transcend drug development. Our hope is that this perspective will help guide investigators to consider the experience with the treatment of TGCT in an effort to duplicate its success in other adult solid cancers.

## BIOLOGIC INSIGHTS

Patients and clinicians alike benefit from understanding the developmental biology of TGCT. Pioneering work by Gillman and Witschi [7,8], connected the embryology of germ cells in both male and female with the functionality of individual cellular components [7,8]. The clinicopathologic studies of Dixon and Moore [6] succeeded in closing the gap between germ cell development and germ cell cancers.6 Seminal observations of embryonic, extraembryonic, and gonadal germ cells and their ontogenetic relationship with TGCT (e.g., embryonal carcinoma, choriocarcinoma, and seminoma, respectively) contributed to the classification of TGCT by Friedman and Moore [1], followed by Teilum with his addition of yolk sac tumor [9], and by Oosterhuis and Looijenga as the current version stands today [10].

Stevens [11] also provided invaluable biological insights into TGCT when he observed more than 600 spontaneous testicular teratomas in strain 129 mice, but only one ovarian teratoma during the same time period. Although male and female strain 129 mice had the same genetic defects, their cellular fate and malignant potential could not have been more different [11]. Stevens [12] also demonstrated that different germ cells in the stem cell hierarchy have different malignant potential. He proved a stem cell rather than a genetic origin of cancers by showing that mice lacking primordial germ cells failed to develop teratoma despite carrying the putative genetic mutation, i.e., Steel (SL), that would have caused the teratoma [12].

It turns out that what is true in the experimental model of teratoma in strain 129 mice is also true in the epidemiology of TGCT in humans. Tumors of germ cell origin constitute about 95% of testicular cancers, but are relatively infrequent (5%) in ovarian cancers. Several reports indicate that the incidence of malignant GCT in men is about 100x higher than in women (although not as high as the >600x observed in the strain 129 mouse counterparts) [13,14]. Importantly, a susceptibility to malignant GCT formation could be traced to an intrinsic difference in the basic biology of male vs. female germ cells. While male germ cells are premeiotic, female germ cells are arrested in meiosis I [15]. It is of interest whether this difference may account for the disparity in the incidence and virulence of GCT in post-pubertal male versus female patients (and versus pre-pubertal patients).

There is a biological impetus and clinical necessity to categorize patients into therapeutically relevant subgroups with functional inferences to account for clinical observations. In the case of TGCT, epigenetic factors seem to trump genetic markers. For example, pure seminoma is the most common histological subtype of TGCT (about 50%). Seminomas are indolent tumors often infiltrated by an abundance of lymphocytes. They have a predictable pattern of spread to the draining lymph nodes. In contrast, pure choriocarcinoma is rare (<1%). Widespread metastasis, rapid progression, and potential hemorrhage clinically distinguish choriocarcinoma from seminoma. Although seminoma and choriocarcinoma in a mixed TGCT may have the same genetic signature, such as the presence of i(12p), their clinical trajectories and pathological attributes could not be more distinct [16]. In short, the clinicopathological features of TGCT have predictive and prognostic implications beyond their intrinsic genetic makeup.

## CLINICAL LESSONS

Given its curability, a major goal in the treatment of TGCT is to preserve the high cure rate while reducing the therapeutic burden. Another crucial challenge is to identify the remaining < 10% of lethal TGCTs that are refractory to current treatment modalities and may require alternative therapeutic strategies. Both goals require us to further investigate the basic biology of TGCT, such as the origin and nature of its underlying intratumoral heterogeneity [4,16,17], and to better elucidate its clinical implications.

For example, embryonal carcinoma is a life-threatening “poorly differentiated” cancer. Without treatment, it is morbidly fulminant and rapidly fatal. Hence, stage I Non-Seminomatous Germ Cell Tumor (NSGCT) containing pure embryonal carcinoma is at an increased risk for relapse on active surveillance. However, its predictive outcome (likelihood and strength of response to treatment) is actually better than its prognostic indicator (native aggressiveness and lethality of disease) because embryonal carcinoma is an exquisitely chemosensitive tumor. Consequently, high-risk pure embryonal carcinoma (e.g., clinical stage IIIC) is not only very treatable, but also curable with chemotherapy [4].

In contrast, certain yolk sac tumors seem to have a “welldifferentiated” phenotype and to behave like teratoma. Although they are relatively indolent, they are also inherently chemo-resistant. Hence, stage I NSGCT containing yolk sac tumor and teratoma is at a decreased risk for relapse on active surveillance. However, its indolent course belies its potentially lethal nature when a diagnosis is missed or surgery is delayed [4,17] In other words, the prognostic outcome of NSGCT containing yolk sac tumor is perplexingly better than its predictive outcome, as long as a diagnosis is made and surgery is performed in a timely manner before the cancer becomes disseminated and is no longer amenable to eradication with curative intent using systemic treatments, such as chemotherapy.

Similarly, certain embryonal carcinomas, such as those containing polyembryomas, may be primarily chemo-resistant and inherently lethal [18]. Certain yolk sac tumors plus seminoma are also innately platinum-refractory and predisposed to somatic transformation [4]. In addition, a peculiar subtype of HCG-producing TGCT, including that presumed to be pure seminoma with tubular elements or known to be epitheliod cytotrophoblastic tumor, may also prove deadly if managed like a regular TGCT with standard, salvage, and high-dose chemotherapies [19,20]. It is plausible that the use of more intensive and highly toxic chemotherapies for the treatment of such intractable tumors is not only futile but also ill advised.

Therefore, early detection and recognition of putative lethal TGCT subtypes is necessary to further enhance the cure rate and simultaneously improve the therapeutic ratio. These potentially lethal TGCTs are likely to be indolent, giving us a false sense of security when patients remain asymptomatic and seemingly unthreatened [17]. Unfortunately, when it becomes widely disseminated it may no longer be curable because of its latent chemo-resistance. However, most if not all of these phenotypes can be cured by surgery, if it is performed in a timely manner when the tumor is still confined to the testis (stage I) or within the retroperitoneum (clinical stages II A and B). We forewarn that conventional modalities using additional chemotherapies followed by surgery may further delay the obligatory retroperitoneal lymph node dissection and miss a window of opportunity to cure these patients before their TGCTs become extensively metastatic.

## THERAPEUTIC STRATEGIES

TGCT provides an excellent example of the value of therapy versus drug development in cancer care. As mentioned above, TGCT is a curable cancer. Many people may attribute its high cure rate to chemotherapy. Although chemotherapy (i.e., drug development) has most certainly contributed to our success in curing TGCT, the high cure rate depends on how much we know about the disease and understand about its treatments.

To put everything in perspective, in 1946, about 90% of patients with metastatic TGCT died within one year of diagnosis [1] By 2016, over 90% of patients with the same diagnosis were cured [4] Thanks to chemotherapies such as vinblastine (approved by the FDA in 1965), Bleomycin (in 1973), and cisplatin (in 1978), the cure rate was already around 60% by 1977 when a regimen known as PVB that combined the three drugs was used to treat testicular cancer [21]

As far as drug development is concerned, randomized trials using newer drugs such as etoposide (approved by the FDA in 1983), ifosfamide (in 1988), and paclitaxel (in 1972) have demonstrated clinical benefits. However, it is also evident that the improvement in overall survival time from 1977 until 2016 is additive rather than multiplicative, and the numbers simply do not add up.

Hence, BEP (using etoposide instead of vinblastine) was statistically better than PVB, but the complete response and twoyear survival rates were similar between the two regimens [22] Subsequently, VIP (using ifosphamide instead of etoposide) [23,24] and a dose-dense regimen (adding paclitaxel) [25] were shown to be equal to BEP.

Therefore, the improved cure rate of TGCT from about 60% in 1977 to over 90% today is not because we have designed better drugs (we have not), but because we have learned how to use the same drugs in the right patients under the right situations. In other words, for patients with early seminoma, we radiate it, and for those with residual teratoma, we surgically remove it. In fact, to improve the cure rate of patients with stage I TGCT, we paradoxically should use less chemotherapy, i.e., fewer drugs.

Since most randomized trials using new drugs or new combinations of drugs have provided incremental rather than exponential survival benefits, and there has been a paucity of novel effective drug development for the treatment of TGCT, the numbers suggests that the overall survival improvement over the past 40 years or so must be due to other reasons beyond drug development. Although drug discovery undoubtedly played an important role in the improved cure rate for patients with TGCT, we surmise that the high cure rate observed in the treatment of patients with metastatic TGCT in the recent past is likely due to improved therapy development, just as much as (or perhaps even more than) novel drug development. The totality of therapy development has included the development of effective anti-emetic strategies, the availability of growth factors, improved surgical and anesthetic techniques, improved postoperative recovery strategies, refined staging incorporating cross sectional imaging, guideline based follow up strategies, and reporting on the continued follow up of the many men that untrusted clinicians in their participation in clinical trials.

Therefore, a critical difference between therapy and drug development in cancer care is whether we have acquired and applied the proper biologic insights (e.g., genetic defects vs. cellular context) and clinical lessons (e.g., genetic targets and pathways vs. cellular networks and tumor subtypes). To cure a complex solid tumor in an efficient and effective manner, we need to go beyond drug development and invest in therapy development in which we target not just the genetic targets and pathways but also the cellular networks and tumor subtypes in an individual person.

## CONCLUSION

In summary, we may cure more patients without resorting to more drugs or new drugs when we apply smarter multimodal therapy based on better biologic insights, as well as improved clinical acumen. This is the meaning of personalized cancer care. In practice, this is the implication of therapy versus drug development in improving the overall outcome and cure rate of patients with TGCT in general and refractory TGCT in particular.
